# Virulence of Cholera Toxin Gene-Positive *Vibrio cholerae* Non-O1/non-O139 Strains Isolated From Environmental Water in Kolkata, India

**DOI:** 10.3389/fmicb.2021.726273

**Published:** 2021-08-20

**Authors:** Eizo Takahashi, Sadayuki Ochi, Tamaki Mizuno, Daichi Morita, Masatomo Morita, Makoto Ohnishi, Hemanta Koley, Moumita Dutta, Goutam Chowdhury, Asish K. Mukhopadhyay, Shanta Dutta, Shin-Ichi Miyoshi, Keinosuke Okamoto

**Affiliations:** ^1^Collaborative Research Center of Okayama University for Infectious Diseases in India, NICED-JICA Building, Kolkata, India; ^2^Department of Health Pharmacy, Yokohama University of Pharmacy, Yokohama, Japan; ^3^Graduate School of Medicine, Dentistry and Pharmaceutical Sciences of Okayama University, Okayama, Japan; ^4^Department of Bacteriology I, National Institute of Infectious Diseases, Tokyo, Japan; ^5^National Institute of Cholera and Enteric Diseases, NICED-JICA Building, Kolkata, India

**Keywords:** *Vibrio cholerae*, NAG Vibrio, cholera toxin, virulence, environmental water, gene analysis

## Abstract

Cholera toxin (CT)-producing *Vibrio cholerae* O1 and O139 cause acute diarrheal disease and are proven etiological agents of cholera epidemics and pandemics. On the other hand, *V. cholerae* non-O1/non-O139 are designated as non-agglutinable (NAG) vibrios and are not associated with epidemic cholera. The majority of NAG vibrios do not possess the gene for CT (*ctx*). In this study, we isolated three NAG strains (strains No. 1, 2, and 3) with *ctx* from pond water in Kolkata, India, and examined their pathogenic properties. The enterotoxicity of the three NAG strains *in vivo* was examined using the rabbit ileal intestinal loop test. Strain No. 1 induced the accumulation of fluid in the loop, and the volume of fluid was reduced by simultaneous administration of anti-CT antiserum into the loop. The volume of fluid in the loop caused by strains No. 2 and 3 was small and undetectable, respectively. Then, we cultured these three strains in liquid medium *in vitro* at two temperatures, 25°C and 37°C, and examined the amount of CT accumulated in the culture supernatant. CT was accumulated in the culture supernatant of strain No.1 when the strain was cultured at 25°C, but that was low when cultured at 37°C. The CT amount accumulated in the culture supernatants of the No. 2 and No. 3 strains was extremely low at both temperature under culture conditions examined. In order to clarify the virulence properties of these strains, genome sequences of the three strains were analyzed. The analysis showed that there was no noticeable difference among three isolates both in the genes for virulence factors and regulatory genes of *ctx*. However, vibrio seventh pandemic island-II (VSP-II) was retained in strain No. 1, but not in strains No. 2 or 3. Furthermore, it was revealed that the genotype of the B subunit of CT in strain No. 1 was type 1 and those of strains No. 2 and 3 were type 8. Histopathological examination showed the disappearance of villi in intestinal tissue exposed to strain No. 1. In addition, fluid accumulated in the loop due to the action of strain No. 1 had hemolytic activity. This indicated that strain No. 1 may possesses virulence factors to induce severe syndrome when the strain infects humans, and that some strains of NAG vibrio inhabiting pond water in Kolkata have already acquired virulence, which can cause illness in humans. There is a possibility that these virulent NAG vibrios, which have acquired genes encoding factors involved in virulence of *V. cholerae* O1, may emerge in various parts of the world and cause epidemics in the future.

## Introduction

Cholera, which causes severe acute diarrheal disease, is a major public-health burden in many developing countries around the world (Harris et al., 2012). Cholera disease is caused by *Vibrio cholerae*. There are many serogroups of *V. cholerae* which ubiquitously inhabits aquatic environments, but *V. cholerae* which cause outbreaks of cholera disease leading to endemic and pandemic outbreaks is limited to only *V. cholerae* serogroups O1 and O139 producing cholera toxin (CT) (Ceccarelli et al., 2019). On the contrary, *V. cholerae* serogroups non-O1/non-O139, which are designated as non-agglutinable (NAG) vibrios, have not caused endemic and pandemic outbreaks, although these bacteria have caused sporadic infections (Morita et al., 2020; Vezzulli et al., 2020). The majority of NAG vibrios do not possess the gene for CT (*ctx*) (Li et al., 2014; Schirmeister et al., 2014; Trubiano et al., 2014).

Cholera pandemics caused by *V. cholerae* O1 have happened six times in the past, and a seventh pandemic is ongoing (Weill et al., 2017). The causative strains from the first to sixth pandemics were classical type *V. cholerae* O1, and that of the seventh pandemic is the El tor type of *V. cholerae* O1. It has been shown that the first six pandemics originated on the Indian subcontinent, and seventh pandemic started in Indonesia in 1961. However, it has been reported recently that the original *V. cholerae* O1 of the seventh pandemic occurred in West Bengal (Hu et al., 2016). Moreover, recent genomic analysis has shown that the seventh pandemic is divided into three groups, designated as waves 1, 2, and 3, respectively. *V. cholerae* O1 in each wave possess unique genetic features (Mutreja et al., 2011; Moore et al., 2014).

It has been reported that global dissemination of every cholera pandemic originated from the Bay of Bengal area (Kaper et al., 1995; Faruque et al., 1998). Similarly, it has been shown that *V. cholerae* O139, which was identified as a novel strain causing a cholera epidemic in 1992–1993 in India, was also discovered in the Bay of Bengal area and spread rapidly throughout India and Asia in the early 1990s (Ramamurthy et al., 1993). Judging from these reports, it is thought that the region around the Bay of Bengal, in which Kolkata is located, is not only the endemic area of cholera but also the place of origin of new virulent *V. cholerae.* Therefore, a comprehensive survey of new virulent *V. cholerae* derived from both clinical sites and environmental sites in Kolkata will be important for the management of endemic and pandemic outbreaks of cholera.

Investigations on *V. cholerae* living in environmental water in Kolkata and the nearby regions are limited. In this study, we carried out an examination of *V. cholerae* inhabiting environmental water in the area of Kolkata, India, and isolated unique NAG vibrios harboring *ctx*, and the pathogenicity of these NAG vibrios was investigated.

## Materials and Methods

### Bacterial Strains and Medium

*Vibrio cholerae* N16961, O1 biovar El Tor (NCBI, Taxonomy ID: 243277), was used as standard strain for *V. cholerae* O1 in this study (Heidelberg et al., 2000). *Vibrio cholerae* isolated in this study were grown in Luria-Bertani medium (LB) at 37°C with shaking and stored at −80°C as a glycerol stock solution.

### Isolation of *V. cholerae* Possessing the Gene for the A subunit of CT From Environmental Water

During May 2012 to May 2017, environmental water sample was collected regularly a couple of times a month throughout the year from 3 ponds in the vicinity of Salt Lake of Kolkata, India. Contamination of filth into these ponds often occurred. The water in these ponds was never used for drinking, but was used for fish farming and washing. The environmental water was collected in sterilized glass sampling bottles (Shibata Scientific Technology LTD., Japan). A portion of the environmental water collected was centrifuged at 5,000 × *g* for 15 min at 25°C and pellet was recovered and suspended in 1/10 volume of sterilized phosphate-buffered saline (PBS). One hundred μL of bacterial suspension and the original environmental water were spread onto thiosulphate citrate bile-salt sucrose (TCBS) agar plates (Eiken Chemical Co., Japan). As it has been shown that some of *V. cholerae* taking vegetative form in environmental water enter into viable but non-culturable state (VBNC state) under certain conditions, though the conditions have not yet been elucidated (Senoh et al., 2014). The bacteria entered into VBNC state cannot grow on agar medium with normal composition, but can grow on the agar medium supplemented with catalase (Mizunoe et al., 2000; Imamura et al., 2015). So, we used TCBS agar medium supplemented with catalase (final concentration 100 U/mL) to detect *V. cholerae* entered into VBNC state.

We inoculated our water sample onto TCBS agar medium with and without catalase and cultured for 18 h at 37°C, all sucrose-fermenting yellow colonies yielded on these plates were picked up and inoculated onto enhanced selectivity (ES) vibrio agar plate (Eiken Chemical Co., Japan). After cultivation for 18 h at 37°C, typical *V. cholerae*-like bacteria grown in the plate were tested the possession of the gene of outer membrane protein W (*ompW*) of *V. cholerae* by PCR using specific primers listed in Table 1 (Nandi et al., 2000). The strains that exhibited DNA bands for *ompW* were regarded as *V. cholerae*. Then, the presence of the gene for A subunit of CT (*ctxA*) and the toxin-coregulated pilus A gene (*tcpA*) encoding subunit A of *V. cholerae* toxin-coregulated pili (TCP) were examined by PCR using the specific primers listed in Table 1. The nucleotide sequence of the 16S ribosomal RNA (rRNA) gene of the isolates used for further analyses in this experiment was determined to confirm the species of these bacteria.

**TABLE 1:** Primers used in this study.

Primer	Gene	Nucleotide sequence	Annealing temp. (°C)	Size of amplicon	Reference or source
**For detection of *V. cholerae* with *ctxA* genes in environmental water**					
ompW-01	*ompW*	caccaagaaggtgactttattgtg	64°C	588 bp	1*
ompW-02		gaacttataaccacccgcg			
ctxA-01	*ctxA*	ctcagacgggatttgttaggcacg	64°C	302bp	2**
ctxA-02		tctatctctgtagcccctattacg			
tcpA-01	*tcpA*	agaagaacacgataagaaaaccg	64°C	124 bp	This study
tcpA-02		cgaatcaatcgcacgctg			
**For sequencing of 16S rRNA gene**					
Universal 27F	16S *rRNA*	agagtttgatcctggctcag	55°C	1,505 bp	3***
Universal 1392R		ggttaccttgttacgactt			

1*, Nandi et al. (2000).

2**, Keasler and Hall (1993).

3***, Weisburg et al. (1991).

### Determination of Serogroup (O-antigen)

The serogroup of the isolates was determined by immunoagglutination with specific antisera. For the determination of serogroups O1 and O139, we used commercially available antisera (Denkaseiken, Tokyo, Japan). Serogroups other than O1 and O139 were determined using 206 O group-specific serum supplied by National Institute of Infectious Diseases of Japan (Yamai et al., 1997).

### Whole Genome Sequencing of NAG Vibrio Possessing *CtxA*

Genomic DNA of the objective NAG strains was extracted from cells, cultivated overnight in LB medium at 37°C with shaking, using a DNeasy Blood & Tissue Kit (Qiagen, Hilden, Germany) in accordance with the manufacturer’s protocol. Illumina libraries of these DNA sequences were prepared using an Illumina DNA Prep Kit (Illumina, Inc. San Diego, CA, United States) and sequencing with HiSeq 2500 (Illumina) or MiSeq (Illumina) sequencers. *De novo* genome assemblies were constructed using CLC Genomics Workbench (Qiagen) and a multicontig draft genome was prepared. Nucleotide sequence data were submitted to the DDBJ Sequenced Read Archive (DRA), and each accession number is listed in Table 2.

**TABLE 2:** Date of isolation, serogroup of strain, genotype of *ctxB*, and accession number of genome.

Strain	Data of isolation	O serogroup	*ctxB* genotype	Accession number
No. 1	June, 2013	O124	B1	DRR296286
No. 2	Oct., 2013	O152	B8	DRR296285
No. 3	July, 2015	Undetermined	B8	DRR296287

### Homology Research

The genes corresponding to each factor in the strains were obtained from their genomic sequences using Genetyx Mac Ver. 19 (Genetyx Corp, Tokyo, Japan). The nucleotide sequence of the reference gene of each factor was cited from GenBank. The accession number and locus tag used are shown in Table 3. Most of reference genes were cited from the genome of *V. cholerae* N16961 strain (Accession No. NC_002505 for chromosome I and NC_002506 for chromosome II) and other genes that were absent in the genome of *V. cholerae* N16961 strain were cited from each reference listed in Table 3.

**TABLE 3:** Virulence gene and regulator genes of *ctx* in the strains analyzed in this study.

	Identity (%)		
	
	Strain No. 1	Strain No. 2	Strain No. 3
**Gene (accession No. and locus of reference gene)**			
*ctxAB* (NC_002505, VC1456, 1457)	99.8% (1146/1148)	99.7% (1144/1148)	99.7% (1144/1148)
*tcpA* (NC_002505, VC0828)	75.3% (508/675)	74.2% (501/675)	76.7% (518/675)
*zot* (NC_002505, VC1458)	99.0% (1188/1200)	98.9% (1187/1200)	99.1% (1189/1200)
*ace* (NC_002505, VC1459)	98.0% (288/294)	99.3% (292/294)	99.3% (292/294)
*hapA* (NC_002506, VCA0865)	98.3% (1799/1830)	99.1% (1814/1830)	100.0% (1830/1830)
*hlyA* (NC_002506, VCA0219)	97.3% (2167/2226)	98.2% (2185/2226)	99.96% (2225/2226)
*rtxA* (NC_002505, VC1451)	Deletion *	98.4% (13460/13677)	Deletion **
*chxA* (NZ_KQ4110624, A55_RS07670)	N.D.	N.D.	N.D.
*stn* (M85198 M36061)	N.D.	N.D.	N.D.
**TTSS (accession No. of reference gene: DQ124262)**			
Gene of component of TTSS (locus tag)			
*VcsN2* (NT01VC2327)	99.2% (1253/1263)	99.1% (1252/1263)	N.D.
*VcsC2* (NT01VC2328)	98.8% (1440/1458)	99.5% (1450/1458)	N.D.
*VcsT2* (NT01VC2330)	98.7% (675/684)	98.8% (676/684)	N.D.
*VcsR2* (NT01VC2331)	96.9% (654/675)	98.7% (666/675)	N.D.
*VcsQ2* (NT01VC2334)	98.1% (212/216)	98.1% (212/216)	N.D.
*VcsU2* (NT01VC2339)	99.3% (1046/1053)	99.3% (1046/1053)	N.D.
*VcsV2* (NT01VC2340)	99.2% (1869/1884)	99.5% (1874/1884)	N.D.
*VspD* (NT01VC2345)	99.1% (1041/1050)	99.3% (1043/1050)	N.D.
*VcsJ2* (NT01VC2348)	97.9% (523/534)	97.9% (523/534)	N.D.
Regulatory gene of *ctx* (accession No. and locus of reference gene)			
*toxT* (NC_002505, VC0838)	100% (831/831)	98.8% (821/831)	98.8% (821/831)
*toxRS* (NC_002505, VC0826, VC0827)	99.5% (1409/1416)	98.6% (1396/1416)	100% (1416/1416)
*tcpPH* (NC_002505, VC0983, VC0984	99.2% (1053/1061)	98.0% (1040/1061)	97.7% (1037/1061)
*hapR* (MF099991)	99.4% (647/651)	99.5% (648/651)	99.2% (646/651)

N.D., Not detected DNA fragment whose sequence was homologous to that of the reference gene. *, The region covering from nucleotide 2,603 to 11,789 of the reference sequence was lost in the gene of the corresponding strain. **, The regions covering from nucleotides 3,028 to 3,198 and 11,332 to 13677 of the reference sequence was lost in the gene of the corresponding strain.

### Production of CT in Liquid Medium

To examine the ability of *V. cholerae* isolates to produce CT, we cultivated the strains in AKI medium (Iwanaga and Yamamoto, 1985). The strain was pre-cultured in LB medium overnight at 37°C with shaking, and 0.2 mL of the pre-culture was inoculated into 10 mL of AKI medium in a 250 mL flask. Two culture suspensions were prepared and cultured statically for 24 h at 25°C and at 37°C, respectively. After cultivation, the culture supernatant was recovered by centrifugation at 20,000 × *g* for 5 min at 4°C. The concentration of CT in culture supernatant was quantified using the GM1-ganglioside ELISA method reported by Kanjilal et al. with a slight modification (Kanjilal et al., 2010). Briefly, a 96-well microplate was coated with monosialoganglioside-GM1 (Sigma-Aldrich, St. Louis, MO, United States) (1 μg/mL in PBS containing 60 mM Na_2_CO_3_) by incubating at 37°C for 4 h. Then, the plate was washed three times with PBS containing 0.05% Tween-20 (PBST). For blocking, the wells were treated with 2% bovine serum albumin in PBS containing 60 mM Na_2_CO_3_ at 4°C overnight. After washing the wells three times with PBST, 100 μL of sample adequately diluted with PBS was applied to the wells and incubated for 2 h at room temperature. After washing the wells three times with PBST, the well was treated with a 1:20,000 dilution of rabbit anti-CT antibody (Sigma-Aldrich) for 1 h at room temperature. Then, the wells were washed three times with PBST and reacted with 1:5,000 dilution of anti-rabbit horseradish peroxidase-conjugated antibody (Abcam, Cambridge, United Kingdom) for 1 h at room temperature, followed by washing the wells three times with PBST. Subsequently, 100 μL of chromogenic tetramethylbenzidine substrate (Becton Dickinson, Franklin Lakes, NJ, United Kingdom) was applied to wells and the plate was kept at room temperature for a few minutes until color appeared. Then, the reaction was stopped by the addition of an equal volume of 3 M H_2_SO_4_. The optical density was measured at 450 nm. Commercially available CT (Sigma-Aldrich) was serially diluted (0.625–10.0 ng/mL) and used for making a calibration curve for CT in this assay.

### Rabbit Ileal Loop Assay

Rabbit ileal loop assays were performed as described previously, using New Zealand White rabbits (weighing about 2 kg) (Koley et al., 1999). Animals were maintained in specific pathogen-free conditions in ventilated cages in the animal house facility of National Institute of Cholera and Enteric Diseases (NICED).

Rabbits were fasted for 24 h before treatment and were anesthetized with sodium pentobarbital. Then, the abdomen of the rabbits was incised and the intestines were exteriorized through a midline incision. After the intestinal lumen was rinsed three times with saline, the intestine was ligated in order to make ileal loops, into which the sample was injected.

Bacteria were cultivated in LB medium at 37°C for 18 h with shaking. The cells were harvested by centrifugation and suspended in an adequate volume of sterilized PBS to make a bacterial suspension adjusting to 2 × 10^9^ colony forming units (CFUs)/mL. The bacterial samples were mixed with an equal volume of either PBS or anti-CT antiserum or normal serum. One mL of these mixtures containing 1 × 10^9^ CFU was injected to the intestinal loop. After injection, the rabbits were kept for 18 h. Then, the rabbits were sacrificed under anesthesia and the intestinal ileal loops were recovered. The volume of accumulated fluid in the loop (mL) and the length of the ileal loop (cm) were measured, and the fluid accumulation ratio (FA ratio), which was the ratio of the volume of fluid accumulated in the intestinal loop to the length of the loop (mL/cm), was calculated.

### Histopathology of Intestinal Tissue

Histopathological analysis of the intestinal tissues of the loops that were exposed to bacteria was performed. Each ileal intestinal loop separated from the body was immediately incised and put into 10% formalin neutral buffer solution. After incubating at room temperature for few days, the tissue sections were embedded in paraffin, and 2–3-μm thick sections made from paraffin-embedded intestinal segments were stained with hematoxylin and eosin. Stained sections were examined using light microscopy, and digital photographs of the stained tissues were taken.

### Hemolytic Activity of Fluid Accumulated in Rabbit Ileal Loops

The hemolytic activity of fluid accumulated in the ileal loop was examined using the lysis of sheep blood cells. The fluid accumulated in these loops was kept at -20°C until use. Just before the examination of hemolytic activity, the frozen samples were thawed and centrifuged 20, 000 × *g* at 4°C for 5 min to remove contaminating material. Fifty μL of the supernatant was transferred to a hole with a diameter of 5.0 mm that had been made in a sheep blood agar plate. After incubation at 37°C for 18 h, formation of a transparent zone around the well was examined.

### Statistical Analysis

Experiments except for animal experiments were carried out in triplicate, independently. The data are presented as arithmetic means ± standard deviation.

## Results

### Isolation of *V. cholerae* Containing the Gene for CT From Pond Water in Kolkata, India

We collected water samples from ponds in Kolkata and cultured these on TCBS agar plates at 37°C for 18 h. Formation of yellow colony was observed in every samples collected. All yellow colonies formed on TCBS agar plate were inoculated onto ES vibrio agar plates, and the presence of *ompW* of *V. cholerae* in all colonies grown on ES vibrio agar was examined by PCR using the primers listed in Table 1. The number of colonies examined was more than 100,000. The band from *ompW* was detected in samples from more than 10,000 colonies. Subsequent searches for *ctxA* and *tcpA* by PCR showed that 5 strains possessed both *ctxA* and *tcpA*, and 32 strains possessed *tcpA* but not *ctxA*. Homology searches from nucleotide sequences of 16S rRNA gene showed that all 37 strains were *V. cholerae*.

The serogroup of O-antigen of the 5 strains possessing both *ctxA* and *tcpA* was determined. Three of the 5 strains did not react with O1 and O139 antisera, whereas the other two strains reacted with O1 antiserum. The former 3 strains were each isolated from separate samples. On the other hand, the latter two isolates were isolated from the same point on the same day. The two isolates appear to have come from the same patient. However, the patient from which these originated has not been identified.

Our interest was to examine the properties of NAG vibrio possessing genes related to the virulence of vibrios. We selected the 3 *ctx*-positive NAG vibrio strains that did not react with O1 and O139 antisera. We designated these 3 NAG vibrio strains as strains No. 1, 2, and 3, respectively. Then we determined the O-serogrop of 3 NAG vibrios using an immunoagglutination test. The serogroup of strains No. 1 and 2 were O124 and O152, respectively. However, it was impossible to determine the O-serogroup of strain No. 3 due to self-agglutination of the bacteria in the test (Table 2).

### Enterotoxicity of NAG Strains

To examine the enterotoxicity of the NAG strains isolated, we carried out rabbit ileal loop assays. The bacteria that were examined in this assay were *V. cholerae* N16961 and four strains of NAG vibrios, strains No. 1, 2, 3, and A. Strain No. A, which we isolated from environmental water, does not possess both *ctxA* and *tcpA* (data not shown). One mL of bacterial suspension containing 1 × 10^9^ CFU/mL in PBS was injected into each loop of the intestine, and the accumulation of fluid in the loop was examined 18 h after injection of the bacterial suspension. PBS and strain N16961 were used as a negative control and a positive control in the assay, respectively. Strain A was used to get the information about the toxicity of NAG Vibrio which did not possess *ctxA* and *tcpA.*

**FIGURE 1:**
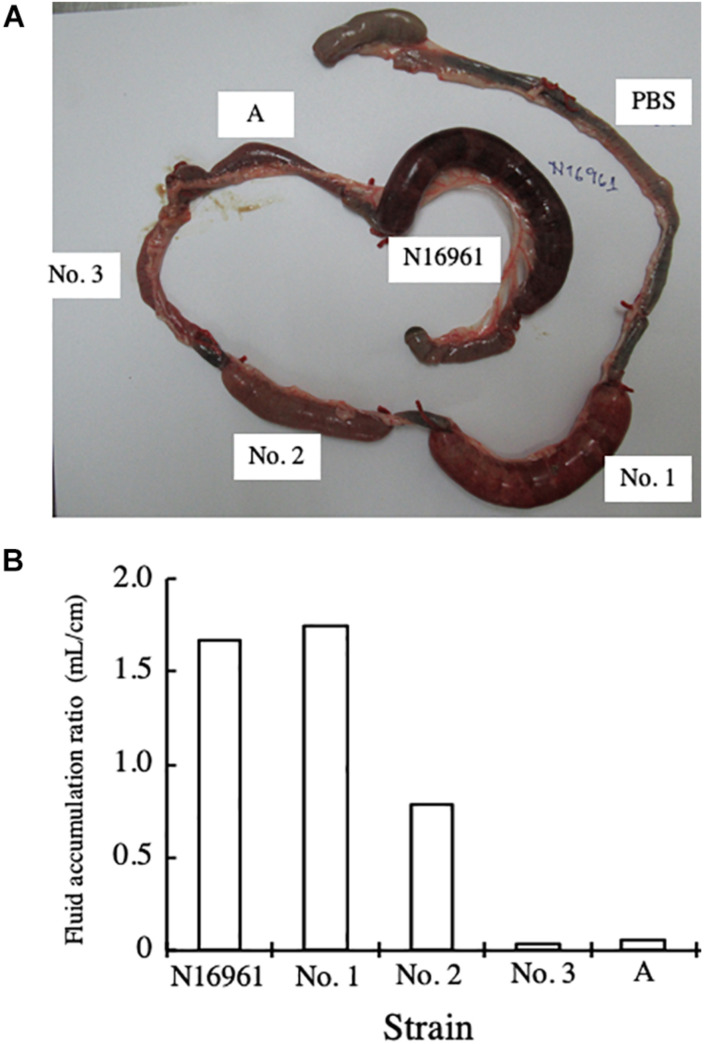
Rabbit ileal loop assay with *V. cholerae.* **(A)** One mL of bacterial suspension containing 1 × 10^9^ CFU/mL in PBS was injected into an intestinal loop in rabbits. The intestine was returned to the abdominal cavity of the rabbit and the rabbit was kept for 18 h. Then, the rabbit was sacrificed under anesthesia and the intestinal ileal loop was taken out. The bacteria examined were *V. cholerae* N16961 and four strains of NAG vibrios (strains No. 1, 2, 3, and A). PBS was used as negative control. **(B)** The volume of fluid accumulated in the loop (mL) and the length of the ileal loop (cm) were measured, and the fluid accumulation ratio, which was the ratio of the volume of fluid accumulated in the intestinal loop to the length of the loop (mL/cm), was calculated.

As shown in Figure 1A, the fluid accumulated in loops administered with N16961. In loops administered with strain No. A and PBS, fluid accumulation was not observed. In the loop administered with strain No. 1, marked fluid accumulation was observed. In the loop administered with strain No. 2, a small but significant volume of fluid accumulated. On the other hand, in the loop administered with strain No. 3, no fluid accumulated.

Then, we collected the fluid accumulated and calculated the FA ratio. As shown in Figure 1B, the FA ratios obtained supported the results of the visual observations.

**FIGURE 2:**
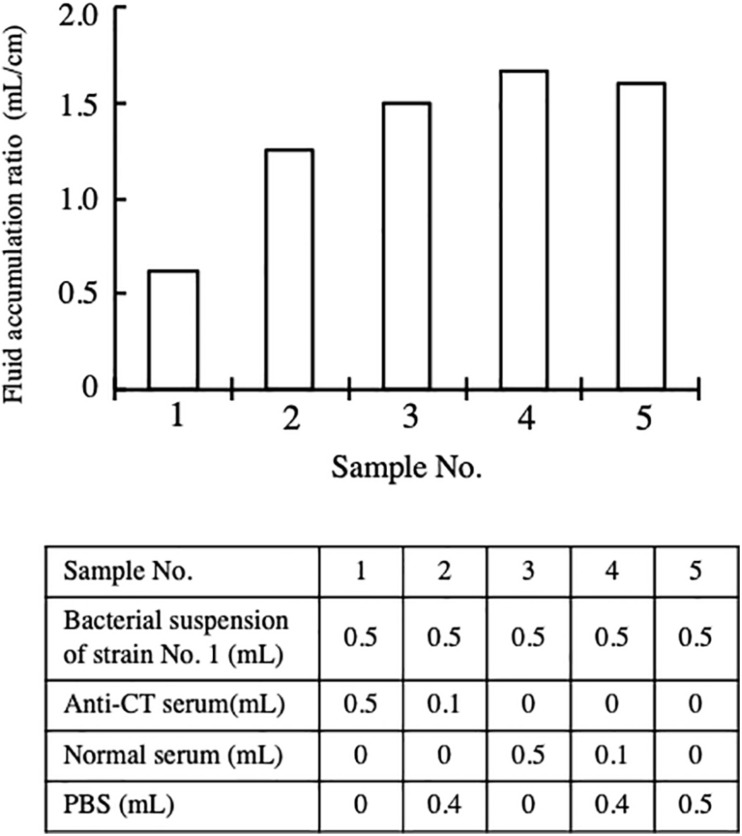
Inhibitory effect of anti-CT serum on the enterotoxicity of strain No. 1. Intestinal ileal loops injected with bacterial suspension of strain No. 1 with or without serum. 0.5 mL of bacterial suspension containing 1 × 10^9^ CFU of bacterial cells was mixed with anti-CT serum or normal serum as shown in the table in the figure. The ileal intestinal loop test using these prepared samples was carried out in the same way in the assay for Figure 1. Fluid accumulation ratio induced by a suspension with different compositions is presented. The fluid accumulation ratio was evaluated as the volume of the fluid accumulated (mL) divided by the length of the ileal loop (cm).

Subsequently, we investigated the contribution of CT to the enterotoxicity of strain No. 1. A bacterial suspension of strain No. 1 containing 2 × 10^9^CFU/mL in PBS was prepared. The bacterial suspension was mixed with an equal volume of serum and PBS. The composition of the mixtures prepared is described in the table in Figure 2, and 1 mL of each mixture was administered into the intestinal loop. The fluid accumulated in the loops was measured 18 h after administration. Fluid accumulated in every loop in which strain No. 1 was administered, although there was difference in the volume accumulated. The FA ratio of these loops was calculated (Figure 2). As shown in the results from samples 3 and 4, fluid accumulation by strain No. 1 was not reduced by the addition of normal serum. However, the FA ratio was reduced by the addition of anti-CT serum. The reduction was dose dependent on the volume of anti-CT serum added to the sample (samples 1 and 2 in Figure 2). When the bacterial suspension of strain No. 1 was mixed with an equal volume of anti-CT antiserum (sample No. 1), the FA ratio caused by the sample was 0.63, which meant that anti-CT serum sufficiently suppressed the enterotoxicity of strain No. 1. This result showed that CT is mainly responsible for the accumulation of fluid caused by strain No. 1.

### Production of CT by NAG Vibrio Strains *in vitro*

To determine the productivity of CT from the isolated NAG strains, CT content in the culture medium was examined.

**FIGURE 3:**
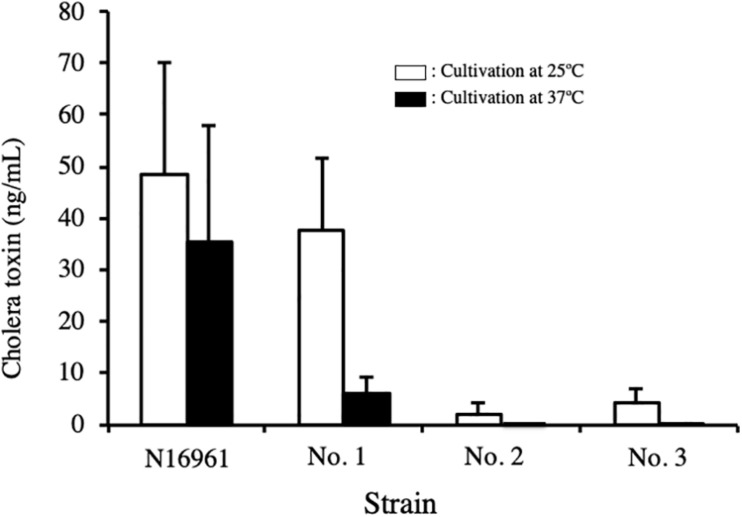
Concentration of cholera toxin in the culture supernatant of *V. cholerae* N16961 and environmental *V. cholerae* non-O1/non-O139 with the *ctx* gene. *V. cholerae* N16961 and three *V. cholerae* non-O1/non-O139 isolates, strains No. 1, 2, and 3 were cultured statically in AKI medium at 25 C and 37°C for 24 h. The concentration of cholera toxin in culture medium was measured using GM1-ELISA. The value for each sample is shown as the mean of three independent replicates. Data are shown as mean + SD. The data from cultures at 25 C and at 37 C are represented with open and solid bars, respectively.

The three NAG strains isolated and *V. cholerae* N16961 were cultured in AKI medium for 24 h at 25°C and 37°C, and the concentration of CT in the culture supernatants was measured by GM1-ganglioside ELISA method using 96-well microplate coated with monosialoganglioside-GM1 as describe in the Materials and Methods. As shown in Figure 3, CT was detected in the culture supernatant of strain N16961, which was used as a CT-positive strain. The amounts of CT in the sample prepared by culturing at 25°C and 37°C was 48.4 ng/mL and 35.6 ng/mL, respectively. Similarly, CT was detected in the culture of strain No. 1 at 25°C. However, the CT level from strain No. 1 cultured at 37°C was very low (Figure 3). However, it is certain that CT is produced even at 37°C. Therefore, it is highly possible that CT is produced in the patient’s body and the CT produced causes diarrhea.

On the other hand, the amounts of CT produced by strains No. 2 and No. 3 were extremely low. Although a small amount of CT was detected in the supernatant of these strains cultured at 25°C, it was not detected in the sample cultured at 37°C (Figure 3).

We considered whether these NAG strains would be able to secrete CT from the cells, although they were capable of synthesizing CT. Therefore, we prepared cell extracts from the cells and measured the CT content in the cell extracts. The cells were collected from the cultures prepared for the experiment in Figure 3 by centrifugation at 15,000 × *g* for 5 min at 4°C and they were suspended in one tenth of the original amount of PBS, and the cell suspensions were disrupted by sonication, and the insoluble material was removed by centrifugation at 20,000 × *g* for 5 min. The recovered supernatant was used as the cell extract, and we measured the concentration of CT. No significant amount of CT was detected in the cell extracts prepared from the 4 strains (N16961 and strains No. 1, 2, and 3) (data not shown).

### Determination of the Genome Sequences of NAG Vibrio Strains

To elucidate the virulence properties of the isolated NAG vibrio strains, we determined genome sequences of these strains using next-generation sequencing methods. Genome sequences of the strains analyzed in this study were deposited in a public database. Accession numbers are listed in Table 2.

### Character of *ctx* and *tcpA*

The sequences of *ctx* gene of these NAG vibrios were highly homologous with that of the reference strain, with an identity level of 99.7% or more (Table 3).

It was shown that the B subunit of CT (CTB) has been divided into 12 groups by differences in its sequence (Kim et al., 2015). The sequences determined showed that the type of CTB of strain No. 1 was *ctx*B1, and those of strains No. 2 and 3 were *ctx*B8 (Table 2).

The identity levels of the sequences of *tcpA* in these NAG strains were low, with a 76.7% homology level or less of with that of reference strain (Table 3).

Judging from the degree of homology of DNA sequence, it is considered that CT produced from these NAG vibrios has sufficient activity. However, since the homology of *tcpA* of isolated NAG vibrios with that of reference strain is in the 70% range, more studies are necessary to clear the activity of TcpA of these NAG vibrios isolated.

### Genes Encoding Other Virulence Factors

Several virulence factors are thought to be related to the toxicity of *V. cholerae* (Figueroa-Arredondo et al., 2001; Ramamurthy et al., 2020), therefore, we searched these genes in 3 NAG strains isolated (Table 3). These virulence factors (such as *zot* encoding zonula occludens toxin, *ace* encoding accessory cholera enterotoxin, *hapA* encoding hemagglutinin, and *hlyA* encoding pore forming cytotoxin) were well conserved in all NAG strains examined (Table 3). It has been revealed from comparative examinations of the sequences of these genes with their reference sequences that nonsense mutations and frameshift mutations were not present in these genes among the three strains examined.

On the contrary, the preservation rate of the genes for *rtxA* (multifunctional autoprocessing repeats-in-toxin), *chxA* (cholix toxin gene), and *stn* (heat-stable toxin (NAG-ST) gene) was low. *chxA* and *stn* were not conserved in the NAG strains examined. *rtxA* was conserved in strain No. 2 but not in the other two strains (Table 3).

### Genes of Factors Constituting the Type III Secretion System

The contribution of the type three secretion system (TTSS) to the pathogenicity of NAG vibrios has been reported (Luo et al., 2013; Zeb et al., 2019), although *V. cholerae* strain N16961 does not contain this system (Heidelberg et al., 2000). As shown in Table 3, genes encoding each protein involved in the TTSS were conserved in the genome of strains No. 1 and 2 but not in strain No. 3.

### Regulatory Genes

The presence of several regulator genes of *ctx* in the isolated NAG strains was examined. The regulatory genes examined were *toxT*, *toxRS, tcpPH*, and *hapR*. These gene products were reported to be involved in the expression of virulence genes, especially in the expression of *ctx* genes (Skorupski and Taylor, 1997; Ramamurthy et al., 2020). As shown in Table 3, all genes were highly conserved (98.0–100% homology) in the genome of all strains examined, and there were no nonsense mutation and no frameshift mutation in these genes.

### Genes of Vibrio Pathogenicity Islands

The strain responsible for the 7th pandemic of cholera is *V. cholerae* O1 biotype El Tor. It is characteristic that the chromosome of *V. cholerae* O1 biotype El Tor consists of core and acquired genomes. The typical acquired genomes have four pathogenicity islands, (i) Vibrio pathogenicity island -1 (VPI-1), (ii) VPI-2, (iii) Vibrio seventh pandemic island-I (VSP-I), and (iv) VSP-II (Pant et al., 2020). We examined the presence of these pathogenicity islands in our isolated NAG vibrios.

The genes constituting VSP-I were not detected in any of the NAG vibrios examined (Table 4).

**TABLE 4:** Genomic islands in the strains used in this study.

Island and gene or cluster (Accession No.) (Locus tag)	Identity (%)		
	
	Strain No. 1	Strain No. 2	Strain No. 3
**VSP-I (Accession No.: NC_002505) (Locus tag: VC0175–VC0185)**			
VC0175-VC0185	N.D.	N.D.	N.D.
**VSP-II (Accession No.: NC_002505) (Locus tag: VC0490–VC0516)** *			
VC0490–VC0498	98.3% (7830/7969)**	N.D.	N.D.
VC0499	N.D.	N.D.	N.D.
VC0501	99.6% (907/911)***	N.D.	N.D.
VC0502–VC0503	N.D.	N.D.	N.D.
VC0504–VC0510	94.1% (2944/3129)	N.D.	N.D.
VC0512–VC0516	N.D.	N.D.	N.D.
**VPI-1 (Accession No.: NC_0025) (Locus tag: VC0817–VC0847)**			
VC0817	99.8% (976/978)	100% (978/978)	100% (978/978)
VC0818	Broken	Broken	100% (681/681)
VC0819–VC0845^* * **^	98.7% (35204/35685)	96.9.0% (34618/35712)	97.3% (34713/35696)
VC0846	99.2.% (235/237)	Broken	Broken
VC0847	99.7% (1265/1269)	99.8% (1267/1269)	99.8% (1266/1269)
VPI-2 (Accession No.: NC_0025) (Locus tag: VC1758–VC1809)*			
VC1758	98.1% (1213/1236)	98.2% (1214/1236)	100% (1236/1236)
VC1759–VC1772	N.D.	N.D.	100% (456/456)
VC1773–VC1786	97.4% (13358/13715)	96.2% (13270/13790)	100% (13788/13788)
VC1788	N.D.	N.D.	100% (696/696)
VC1789–VC1802	N.D.	N.D.	100.00% (10504/10504)
VC1804–VC1807	92.8% (2348/2529)	97.4% (2463/2529)	100.0% (2529/2529)
VC1808	N.D.	N.D.	100.0% (846/846)
VC1809	97.0% (195/201)	97.0% (195/201)	100% (201/201)

N.D., Not detected DNA fragment whose sequence was homologous to that of the reference gene. Broken, Base sequence of the gene is disrupted by the insertion of an unknown gene fragment. *, VC0500, VC0511, VC1780, VC1787, VC1790, and VC1803 were lost from the genome of N16961. **; Insertion of nucleotides generates at two positions. One is the insertion of one base at VC0495. This insertion causes a frameshift mutation. Another insertion occurred outside of the open reading frame. ***; There are mutations with 9-bp and 1-bp insertions. ****, TCP cluster is coded in the loci from VC0824 to VC0837. Fragments of unknown genes were inserted in several boundary positions: Strain No. 1, between VC0817 and VC0818, between VC1772 and VC1773, between VC 1784 and VC1785; Strain No. 2, between VC0817 and VC0818, between VC0824 and VC0825, between VC 1758 and VC1759, between VC1772 and VC1773; Strain No. 3, between VC0837 and VC0838, between VC1788 and VC1789.

VSP-II consisted of the regions from VC0490 to VC0516 in the genome of *V. cholerae* N16961 chromosome I (Heidelberg et al., 2000). Homology analysis revealed that strains No. 2 and 3 do not contain any genes homologous with VSP-II. On the contrary, some regions that are homologous with VC0490–VC0498, VC0501, and VC0504–VC0510, are reserved in the genome of strain No. 1 (Table 4). However, the genes homologous to VC0499, VC0502–VC0503, and VC0512–VC0516, were not detected in the genome of strain No. 1 (Table 4). This showed that strain No. 1 possessed a VSP-II like element in which some loci constituting the canonical VSP-II (VSP-II of *V. cholerae* N16961) were deleted.

VPI-1 contains a gene for a crucial virulence factor of cholerae pathogens, toxin-coregulated pilus (TCP), in the loci from VC0824 to VC0837. These loci were maintained in all NAG strains examined (Table 4). All other loci constituting VPI-1 were present in strain No. 3, but the DNA sequences of VC0818 were disturbed in the genome of strain No. 1, and the DNA sequences of VC0818 and VC0846 in the genome of strain No. 2 were disturbed.

All loci constituting VPI-2 were conserved in strain No. 3, but more than half of the loci that constituted VPI-2, 29 loci of 48 loci of VPI-2, were not detected in strains No. 1 or 2 (Table 4).

### Cytotoxicity of NAG No. 1 Strain in Intestinal Loops

**FIGURE 4:**
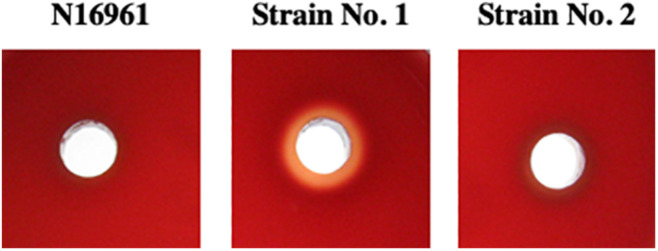
Hemolytic activity of the supernatant of fluid accumulated in intestinal loops due to the action of *V. cholerae*. Fluid that accumulated in the intestinal loops due to the action of *V. cholerae* N16961, strains No. 1 and 2 was centrifuged 20, 000×*g* at 4 C for 5 min and their supernatants were recovered. A 50 μL sample of each supernatant was placed in a well-made in a sheep blood agar plate and incubated at 37°C for 18 h.

We noticed that fluid caused by the administration of No. 1 strain in rabbit ileal loop assays was hemorrhagic red. Therefore, we assumed that strain No. 1 produced toxic factors to induce damage to intestinal tissue *in vivo*. It is known that hemolysin produced by *V. cholerae* often produces cytotoxic action (Mitra et al., 2000). Then, we investigated the hemolytic activity of the accumulated fluid using sheep blood agar plates. As shown in Figure 4, supernatant of fluid accumulated by strain No. 1 exhibited hemolysis, whereas those with N16961 and strain No. 2 did not.

**FIGURE 5:**
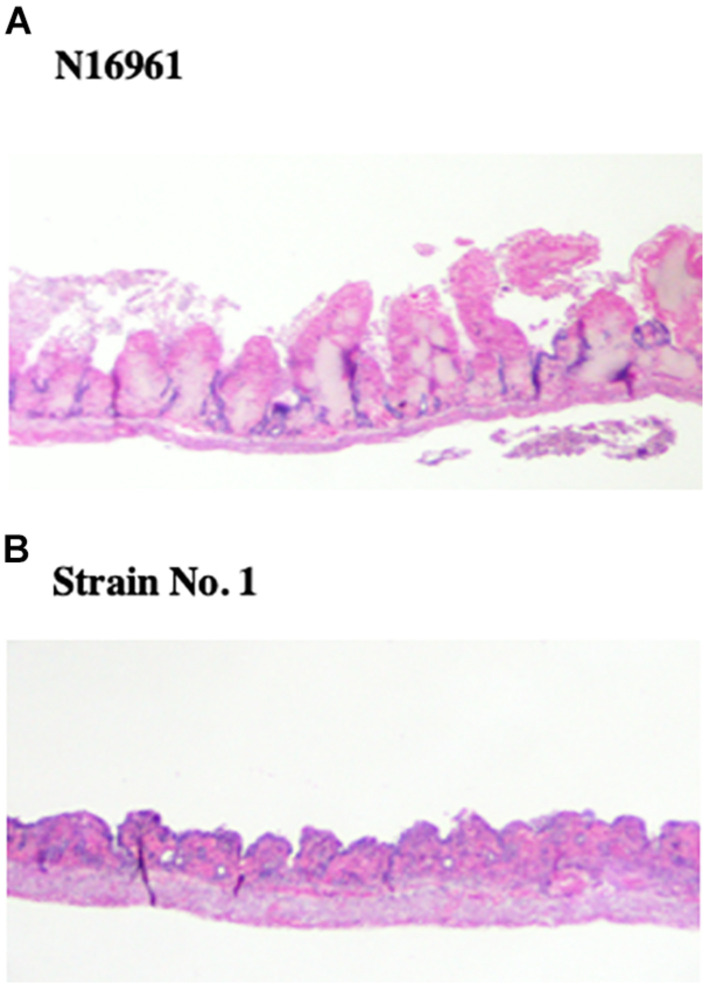
Histopathological examination of intestinal tissues administered *V. cholerae*. From the intestinal loop assay in Figure 1, portions of intestinal tissues from loops administered with N16961 and strain No. 1 were recovered and fixed in 10% formalin neutral buffer solution. Tissues section was prepared as described in the Materials and Methods and stained with hematoxylin and eosin. The samples were examined using light microscopy. **(A)** N16961, **(B)** Strain No. 1.

Subsequently, we examined the intestinal tissues histopathologically from loops exposed to *V. cholerae* N16961 and strain No. 1 in the experiment for Figure 1. The examination showed that many villi were shortened or disappeared in the intestine that was administered with strain No. 1, whereas villi remained without incurring severe damage in intestinal tissue administered with N16961 (Figure 5).

Judging from these results, it was thought that cytotoxic hemolysin was produced by strain No. 1 in the intestinal lumen and the hemolysin produced might damage intestinal tissue, which resulted in the disappearance of villi.

## Discussion

It was shown that almost all NAG vibrios inhabiting the aquatic ecosystem did not possess *tcpA* (Singh et al., 2001; Rajpara et al., 2013). Li et al. reported that 8 strains among 295 NAG vibrios from an aquatic ecosystem in China possessed *tcpA* (Li et al., 2014). In our examination, only 37 NAG vibrio strains among about 10,000 strains isolated from ponds in Kolkata, India, possessed *tcpA*. This showed that the ratio of *tcpA*-positive NAG vibrios to total NAG vibrios in the aquatic ecosystem was very low around the world.

Genes encoding TCP are located in VPI-1, which was introduced by horizontal gene transfer (Manning, 1997; Davis and Waldor, 2003; Ashok et al., 2020). It is considered that the frequency of the transfer of VPI-1 is low in aquatic ecosystems, judging from the low isolation rate of *tcp*-positive *V. cholerae* of all *V. cholerae* in aquatic ecosystems.

In this study, we isolated three NAG vibrios possessing *ctx* from ponds in Kolkata. These three strains also possessed *tcpA*. TcpA is major subunit of TCP, which plays an important role in the process of establishing an infection of *V. cholerae.* For example, TCP is involved in colonization on the surface of the human small intestine, via self-aggregation to protect against host defenses and concentrate the secreted CT (Taylor et al., 1987; Ghasemi et al., 2020). In addition, it has been shown that TCP also acts as a highly specific receptor for the cholera toxin phage (CTXϕ), which can infect non-pathogenic *Vibrio* species and confer virulence by providing genes that encode CT (Faruque et al., 1998).

As described, CTXϕ infects *ctx*-negative *V. cholerae* using TCP as a receptor. CTXϕ cannot bind to *tcp*-negative *V. cholerae*. Therefore, it is usual that *ctx*-positive *V. cholerae* possess *tcp.* In our examination, all *ctx*-positive isolates possessed *tcp.* Judging from the ratio of *tcp-*positive vibrios to all vibrios, that is, 37 strains out of 10,000 strains, it was thought that the opportunity for CTXϕ to come into contact with sensitive *V. cholerae* is low in aquatic ecosystems in Kolkata. From such a low frequency, the rate of appearance of two gene (*tcp* and *ctx*)-positive NAG vibrios becomes extremely low. However, the introduction of *ctx* to *tcp*-positive *V. cholerae* has infrequently occurred in aquatic ecosystems in Kolkata. Thereby, we were able to detect three NAG strains possessing both *tcp* and *ctx.*

These gene products, TcpA and CT, are thought to be involved in the surviving of *V. cholerae* in the human intestinal tract. *V. cholerae* O1 adhering to the intestinal surface cause the secretion of body fluids from the host into the intestinal lumen utilizing CT activity, and this fluid is finally excreted as diarrhea stools. *V. cholerae* O1 can use this fluid as a nutrient source and survive in the intestinal lumen. Without the activity to obtain intestinal fluid, it is hard for *V. cholerae* O1 to survive in the intestinal lumen. Therefore, all *V. cholerae* O1 isolated from diarrheal stool possess these genes encoding CT and TCP. However, it is unclear how CT and TCP are involved in the survival of *V. cholerae* in environmental water. This is a problem to be solved in the future.

The examination of the amount of CT produced outside of the cell showed that the production of CT *in vitro* at 37°C by strain No. 1 was low (Figure 3). This result made us suspicious that the bacteria produced a sufficient amount of CT to cause illness when infecting humans.

**FIGURE 6:**
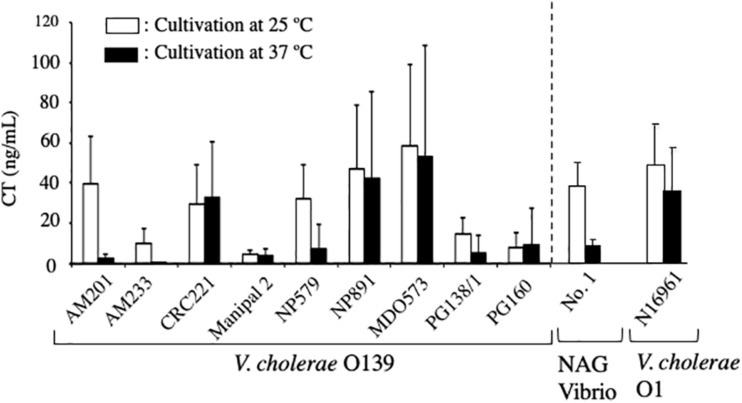
Concentration of cholera toxin in culture supernatants of *V. cholerae* O139, NAG vibrio and *V. cholerae* O1. Nine strains of *V. cholerae* O139 (AM201, AM233, CRC221, Manipal 2, NP579, NP891, MID0573, PG138/1, and PG160), strain No. 1 (NAG Vibrio) and *V. cholerae* N16961 (O1) were cultivated statically in AKI medium at 25 C and 37 C for 24 h and their culture supernatant were recovered by centrifugation. Cholera toxin content in these supernatants were measured using GM1-ELISA and represented with open and solid bars, respectively. The value of each sample is shown as the mean of three independent replicates. Data are shown as mean + SD.

To examine the productivity of CT and the contribution of CT in the infection of NAG Vibrio, we examined the amount of CT produced by toxigenic *V. cholerae* O139 *in vitro*, which was isolated from patients during the pandemic in 1992–1993 in India. These strains were the strains that caused the pandemic at that time. These strains, strain No. 1 and *V. cholerae* N16961 (O1) were cultivated statically in AKI medium at 25°C and 37°C for 24 h and their culture supernatants were recovered by centrifugation. CT content in these supernatants was measured by GM1-ELISA. As shown in Figure 6, the ability of strain No. 1 to produce CT at 25°C was comparable to those of 5 strains of *V. cholerae* O139 (AM201, CRC221, NP579, NF891, and MD0573) and of *V. cholerae* N16961 (O1). Therefore, it seemed that strain No.1 had almost equivalent capacity to produce CT at 25°C with those strains. Moreover, it was interesting that the capacity of the No. 1 strain to produce CT at 25°C was superior to those of the remaining 4 strains (AM233, Manipal 2, PG138/1 and PG160) (Figure 6). As described, all *V. cholerae* O139 examined in Figure 6 are the bacteria isolated as the causative bacteria of the pandemic. Therefore, we think that the No. 1 strain might produce enough amount of CT in the human intestine and cause cholera outbreaks.

In addition, as observed in the No. 1 strain, the amount of CT produced at 37°C was obviously lower than the amount produced at 25°C in 4 strains (AM201, AM233, NP579 and PG138/1). It indicates that the production of CT at 37°C is lower than that at 25°C is a reaction which often occurs in NAG Vibrio. The mechanism for the low production at 37°C has remained unclear.

The reports about the outbreaks of food poisoning by NAG vibrios with *ctx* have been published. These reports show that some NAG vibrios have sufficient pathogenicity to cause illness in humans (Tobin-D’Angelo et al., 2008; Haley et al., 2014; Vezzulli et al., 2020). However, these outbreaks have not developed into a pandemic. It seems that other conditions that are necessary for the occurrence of a pandemic, such as hygienic environment, outflow of sewage, temperature, etc., were not in place in the areas where the food poisoning occurred. Alternatively, these food poisoning NAG vibrios may have been deficient in other factors which are necessary for the generation of the pandemic. If these conditions needed to cause a pandemic were met in the area where food poisoning had spread and/or the genes for other factors which were necessary for the generation of pandemic were transferred to these NAG vibrios, the pandemic might have occurred after a food poisoning incident. If a food poisoning incident occurs due to the infection with strain No. 1, the same situation can be considered. We must pay attention to the existence of such *V. cholerae* that possesses the potential to cause cholera outbreak similar to *V. cholerae* O1 and O139.

We isolated three NAG strains possessing both *tcp* and *ctx*. One of these strains, strain No. 1, showed diarrheagenic activity and cytotoxicity in rabbit intestinal loop assays (Figures 1, 5). Subsequent analyses showed that these two activities were brought about by the actions of CT and hemolysin produced by the bacteria, respectively (Figures 2, 4). As shown in Table 3, strain No. 1 possessed other genes related to virulence such as *zot, ace, hapA, rtxA*, and *hlyA*. It was already reported that hemolysin of NAG vibrios induced fluid accumulation in rabbit ileal loop assays, and that the intestinal mucosa of the loop was damaged by the action of hemolysin (Ichinose et al., 1987). In our experiment, histological changes of mucosa were also observed in loops administered with strain No. 1 and the production of the hemolysin from this strain was confirmed (Figure 4). Therefore, it was likely that hemolysin produced by strain No. 1 caused enterotoxicity in the loop.

As shown in Figure 2, anti-CT serum significantly reduced the amount of fluid accumulated in the loop by strain No. 1. However, the serum could not completely suppress the accumulation by the strain. This indicates that the factor mainly involved in the fluid accumulation by strain No. 1 was CT, however, other factors produced from the bacteria, whose activity was not neutralized with anti-CT serum, contributed to the fluid accumulation. As mentioned, NAG Vibrio hemolysin is thought to be enterotoxic in the intestinal tract. We think that one of other factors is the hemolysin.

In addition, it was indicated that strain No. 1 possessed a TTSS, which has been regarded as a key virulence factor for pathogenicity of NAG vibrios (Table 3) (Luo et al., 2013; Zeb et al., 2019). These findings suggested that strain No. 1 contains virulence factors not only from toxigenic *V. cholerae* O1 and O139 but also from NAG vibrios. If strain No. 1 has a chance to infect to a person, the strain probably would cause severe syndrome in the host due to the action of these two virulence factors derived from CT-positive *V. cholerae* and from NAG vibrios. Then, it is likely that infection with strain No. 1 may spread to people in the vicinity of the patient, as *V. cholerae* O139 once spread in India, Bangladesh, and neighboring countries in the early 1990s (Ramamurthy et al., 1993; Nair et al., 1994).

More notably, concerns over the emergence of new NAG vibrios acquired other virulence properties have been put forward (Vezzulli et al., 2020). These virulence properties might be introduced into NAG vibrios by genetic exchange mechanisms such as horizontal gene transfer and genetic recombination. The changing environment and climate were proposed to be factors in the emergence of new NAG vibrios. The emergence of such virulent NAG vibrios is a threat to humankind. Actually, a new type of NAG vibrio with *ctx* has been isolated from a patient with persistent diarrhea (Kumar et al., 2018). The strain possessed Haitian type CT, which is a type of CT, generated recently from the transfer of the mobile element encoding *ctx*. We must be vigilant against the generation of such highly pathogenic *V. cholerae* from non-O1/non-O139.

Among NAG strains examined in this study, strain No. 1 contained the majority of the locus of VSP-II, but it did not contain the region of VSP-I. It is speculated that the movement of VSP-II to strain No. 1 was carried out by the horizontal transfer events using the ability to excise to a circular intermediate which was reported in *V. cholerae* N16961 (Murphy and Boyd, 2008). Further studies are necessary for the determination of the acquisition mechanism of VSP-II by strain No. 1.

The ileal loop test showed that the enterotoxicity of strain No. 1 was higher than that of the other two strains, strains No. 2 and No. 3 (Figure 4). Strains No. 2 and 3 did not contain the VSP-I and VSP-II regions and strain No. 1 contains VSP-II but not contain VSP-I (Table 4). Therefore, we considered the possibility that VSP-II acts to promote the enterotoxicity of *V. cholerae*, though the consideration is speculative at present. However, the consideration is also supported by the reports described below.

The ongoing seventh cholera pandemic has spawned several novel El Tor variants of *V. cholerae* O1, including the Matlab, Mozambique, altered El Tor, and recently the Haitian variant (Nair et al., 2002, 2006; Faruque et al., 2007; Chin et al., 2011). Among these, the Haitian variant has turned out to be the most deadly strain, affecting around 0.8 million people along with around 9,000 deaths in Haiti (Piarroux et al., 2011). It was also shown that the Haitian variant *V. cholerae* O1 strains isolated in Kolkata produced higher amounts of CT compared with contemporary O1 El Tor variant strains *in vitro* (Naha et al., 2020). In addition, these Haitian variant *V. cholerae* O1 strains manifested higher virulence in an animal model (Ghosh et al., 2019). The study of gene sequences showed that *V. cholerae* isolates from Haiti revealed unique genetic changes in the region of VSP-II (Chin et al., 2011). Other genetic analyses of these highly virulent strains showed that variation in VSP-II has progressed in these Haitian strains (Nusrin et al., 2009; Imamura et al., 2017; Severin et al., 2018). Therefore, it is likely that the gene product(s) of VSP-II may influence the production of CT and the virulence of *V. cholerae* O1.

Our analysis showed that some loci of VSP-II were deleted in VSP-II of strain No. 1 (Table 4). It has been shown that the stability VSP-II is not high and that some loci are often deleted (Haley et al., 2014; Imamura et al., 2017). Studies using strains possessing partially deleted VSP-II, such as strain No. 1, might be useful for analysis of the action of each locus of VSP-II.

Whole genome sequence analysis of El Tor seventh pandemic strains has showed that these strains have been divided into three waves; waves I, II, and III (Harris et al., 2012; Kim et al., 2015; Ramamurthy et al., 2019). Whole genome sequence analysis also showed that CTB is classified into several types by the substitution of specific amino acid residues, although the A subunit of CT was highly conserved (Harris et al., 2012; Ramamurthy et al., 2019). It has been reported that the strain in each wave of pandemics possesses a distinct CTB, and the CTB type is one source to infer the origin of the strain (Harris et al., 2012; Ramamurthy et al., 2019). Therefore, we analyzed the sequence of *ctxB* encoded in our isolates and determined their types (Table 2). The B subunit type in strain No. 1 was 1 (*ctxB1*). *V. cholerae* O1 with *ctxB1* was originally found prior to the seventh pandemic. Subsequently, the El Tor type of *V. cholerae* O1 with *ctxB1* was isolated in the early 1990s and was dominant around 1995 in Kolkata (Raychoudhuri et al., 2009; Kim et al., 2015). It seems that the population of CTX prophage-containing *ctxB1* increased and strain No. 1 isolated in this experiment may have appeared in that period.

At present, it has been shown that CT B genotypes of *ctxB1*, *ctxB2*, *ctxB3*, *ctxB4*, *ctxB5*, and *ctxB6* have been linked with pandemic strains of *V. cholerae* O1 and O139 (Kim et al., 2015). In addition, it has been shown that the genotype of *V. cholerae* O1, which has been isolated recently from all over the world including Haiti, Yemen, Mariupol, etc. are *ctxB7* (Ramamurthy et al., 2019). In contrast, *ctxB8* was originally detected from *V. cholerae* O27, which was isolated from prawn (Li et al., 2002; Kim et al., 2015). Subsequently, *ctxB8* has been detected in novel variants of *V. cholerae* O1 that were isolated from the environment (Zhang et al., 2013; Kim et al., 2015). So far, *V. cholerae* with *ctxB8* has not been isolated from clinical samples. This may mean that the CTXϕ carrying *ctxB*8 may have the property that it does not bind to the pathogenic *V. cholerae* and/or does not grow in these bacteria. Further studies are necessary to resolve the issue.

## Data Availability Statement

The datasets presented in this study can be found in online repositories. The names of the repository/repositories and accession number(s) can be found below: https://www.ncbi.nlm.nih.gov/sra/DRR296286; https://www.ncbi.nlm.nih.gov/sra/DRR296285; and https://www.ncbi.nlm.nih.gov/sra/DRR296287.

## Ethics Statement

The animal study was reviewed and approved by the Institutional Animal Ethics Committee (IAEC) of the National Institute of Cholera and Enteric Diseases with Registration no.: 68/GO/ReBi/S/99/CPCSEA valid 17/7/2024, Approval no: PRO/120/April 2016–March 2019.

## Author Contributions

ET and KO designed the research and wrote the first draft of the manuscript. MM and MO participated in the sequencing experiments. SO and S-IM participated in the analysis of the sequencing data. TM, DM, GC, AM, and SD participated in the isolation of *V. cholerae*. HK and MD participated in the examination of pathogenicity of bacteria. All authors took part in editing the manuscript, read and approved the final version.

## Conflict of Interest

The authors declare that the research was conducted in the absence of any commercial or financial relationships that could be construed as a potential conflict of interest.

## Publisher’s Note

All claims expressed in this article are solely those of the authors and do not necessarily represent those of their affiliated organizations, or those of the publisher, the editors and the reviewers. Any product that may be evaluated in this article, or claim that may be made by its manufacturer, is not guaranteed or endorsed by the publisher.
